# Plasmonic nanomeshes: their ambivalent role as transparent electrodes in organic solar cells

**DOI:** 10.1038/srep42530

**Published:** 2017-02-15

**Authors:** Christian Stelling, Chetan R. Singh, Matthias Karg, Tobias A. F. König, Mukundan Thelakkat, Markus Retsch

**Affiliations:** 1 Physical Chemistry – Polymer Systems, University of Bayreuth, Universitätsstr. 30, 95447 Bayreuth, Germany; 2 Applied Functional Polymers, Macromolecular Chemistry I, University of Bayreuth, Universitätsstr. 30, 95447 Bayreuth, Germany; 3 Physical Chemistry I, Heinrich-Heine-Universität, 40204 Düsseldorf, Germany; 4 Institute of Physical Chemistry and Polymer Physics, Leibniz-Institut für Polymerforschung Dresden e. V., Hohe Straße 6, 01069 Dresden, Germany and Cluster of Excellence Centre for Advancing Electronics Dresden (CFAED), Technische Universität Dresden, 01062 Dresden, Germany

## Abstract

In this contribution, the optical losses and gains attributed to periodic nanohole array electrodes in polymer solar cells are systematically studied. For this, thin gold nanomeshes with hexagonally ordered holes and periodicities (*P*) ranging from 202 nm to 2560 nm are prepared by colloidal lithography. In combination with two different active layer materials (P3HT:PC_61_BM and PTB7:PC_71_BM), the optical properties are correlated with the power conversion efficiency (PCE) of the solar cells. A cavity mode is identified at the absorption edge of the active layer material. The resonance wavelength of this cavity mode is hardly defined by the nanomesh periodicity but rather by the absorption of the photoactive layer. This constitutes a fundamental dilemma when using nanomeshes as ITO replacement. The highest plasmonic enhancement requires small periodicities. This is accompanied by an overall low transmittance and high parasitic absorption losses. Consequently, larger periodicities with a less efficient cavity mode, yet lower absorptive losses were found to yield the highest PCE. Nevertheless, ITO-free solar cells reaching ~77% PCE compared to ITO reference devices are fabricated. Concomitantly, the benefits and drawbacks of this transparent nanomesh electrode are identified, which is of high relevance for future ITO replacement strategies.

Transparent conducting electrodes (TCEs) are inevitable in modern electro-optical devices such as organic light-emitting diodes, touch displays, photodetectors or solar cells. However, materials that combine a high optical transparency and a high electrical conductivity are very rare^1^. The state-of-the-art transparent electrode materials are conducting oxides like indium tin oxide (ITO). Such oxides offer a high transmittance surpassing 90% over the whole visible range, and a low sheet resistance down to 10 Ω/□^2^. However, a lot of effort is put into the development of alternative electrode materials due to the well-known drawbacks of ITO: Besides the scarcity and thus high costs of indium, the conductivity of ITO strongly depends on its thickness. Additionally, ITO is very brittle, limiting the beneficial properties of polymer solar cells, which are their applicability for low-cost roll-to-roll processing onto flexible substrates^3^. The use of carbon nanotubes^4^, thin metal films^5^, solution-processed metal nanowires^6^, metal grids prepared by lithography^7^,^8^, printing^9^ or cracked tamplates^10^,^11^ and metal nanohole arrays^12^,^13^,^14^ have been discussed to replace ITO.

However, carbon nanotube grids still suffer from low stability under ambient conditions, while metal nanowire networks and thin metal films are limited due to their reduced transmittance compared to ITO^7^,^15^. Furthermore, solution processed nanowires exhibit a high resistance at junctions and easily cause electrical short circuits due to wires penetrating through the blocking layer^16^.

A promising alternative to ITO could be periodic metal nanohole arrays, also referred to as nanomeshes. Due to their exceptional optical properties such as extraordinary optical transmittance (EOT)^17^ they have been suggested as transparent conducting electrodes for both inorganic^18^ and organic^19^ solar cells. This is based on their ability to support surface plasmon polaritons (SPP)^20^,^21^,^22^. The role of surface plasmons in the performance of thin film solar cells has extensively been studied in the past years^23^,^24^,^25^,^26^,^27^,^28^. Surface plasmons have been proposed to trap incident light at the metal/semiconductor interface and thus enhance the light absorption in photovoltaic devices due to an increased interaction time between the light and the active layer and an enhanced field intensity in the device. Yet, there is still a controversy whether or not nanomesh plasmonic electrodes are better suitable candidates as TCE by combining both their transmission and plasmonic properties. Unambiguous and consistent reports on clear plasmonic contributions, exceeding the improvements obtainable by simple scattering mechanisms are scarce.

Usually the better suitability of such nanomeshes is demonstrated just by testing them in devices and comparing with ITO references. An increase in the power conversion efficiency was shown for a silver nanohole array in a small-molecule solar cell compared to an unpatterned silver film and ITO as reference^29^. In that case, the active layer was in direct contact with the 12 nm thick nanohole array (400 nm period), which was prepared by colloidal lithography. The enhanced efficiency of such devices can originate from plasmonic enhancement in direct proximity to the nanomesh as well as from an increase in the interfacial area between hole and electron conductor.

Beside their application as transparent electrodes, the introduction of nanohole arrays as back reflectors in organic solar cells resulted in an enhanced device absorption by coupling of the light to SPP modes in experimental^30^ and numerical studies^31^,^32^,^33^ for ultrathin active layers of P3HT blended with fullerenes.

Some work has been done on integrating nanohole arrays as transparent electrodes for polymer solar cells. Randomly ordered nanoholes showed high losses in the J_SC_ due to the limited transmittance of the electrodes^34^,^35^. Nevertheless, the IPCE measurements hint at a contribution of SPPs to the device performance. Numerical studies show the excitation of a guided mode localized in the P3HT:PC_61_BM active layer at long wavelengths beyond the absorption edge of P3HT^19^.

Zhu *et al*. used colloidal lithography to fabricate an electrode with a thickness of 18 nm, a hole diameter of 357 nm and an initial particle diameter of 430 nm^36^. The reduction in J_SC_ compared to the state-of-the-art electrode ITO was attributed to the reduced transparency.

Morfa *et al*. described the influence of the hole diameter on the sheet resistance of bare Ag nanohole arrays prepared by nanosphere lithography followed by plasma etching to reduce the initial particle diameter^37^. Short etching times yielded high sheet resistances due to a discontinuous metal structure or wire thicknesses in the range of the mean free path of the electrons in silver. Longer etching times, on the contrary, resulted in a rapidly decreased transmittance. This demonstrates a fundamental dilemma and it is not clear up to now whether periodically structured metal electrodes can provide a net improvement.

For polymer solar cells, Chou *et al*. reported higher efficiencies for a subwavelength square array of nanoholes^38^. The bulk heterojunction devices with P3HT:PC_61_BM as active layer showed a drastic decrease in reflectance when incorporating the nanohole array into the device. By choosing an appropriate active layer thickness, which supports a coupling between the front and back electrode, the incident light was confined in the active layer of the device. Therefore, the absorption of the device was radically boosted, exceeding even the efficiency of the reference ITO device. This effect was not maintained for the bare array or devices with a PMMA layer of the same thickness.

Overall, the distinct optical loss mechanisms related to parasitic absorption and reflectance for nanohole array electrodes have not been studied systematically and clarified properly. It is therefore not possible to evaluate the advantages and shortcomings of periodic nanohole electrodes on a broader scale. This, however, will be of great importance, if nanohole electrodes were to be considered as ITO replacements in current and future optoelectronic devices.

Therefore, in this paper, we focus on two main aspects, which are decisive for light management in any solar cell. First, we vary the periodicity of gold nanohole array from 202 nm up to 2560 nm and thereby fully address the relevant optical range from visible to near-infrared. Secondly, we investigate the interplay of different optical and electrical properties of these electrodes in photovoltaic devices with two different and well-known bulk-heterojunction photoactive layers, P3HT:PC_61_BM and PTB7:PC_71_BM in an inverted solar cell device architecture. P3HT:PC61BM was selected as it is the most studied material composition in polymer photovoltaic research^39^. On the other hand, up to 9% power conversion efficiency has been reported with a highly optimized PTB7:PC_71_BM system^40^. We have purposefully selected these material systems as universal prototypes in which the absorption edge of donor polymers varies from the visible to the near-infrared region. We rationalize our findings by finite-difference time-domain (FDTD) simulations to provide a full understanding of the optical properties and assess the potential of metal nanomeshes as transparent conducting electrodes.

## Results and Discussion

**Figure 1:**
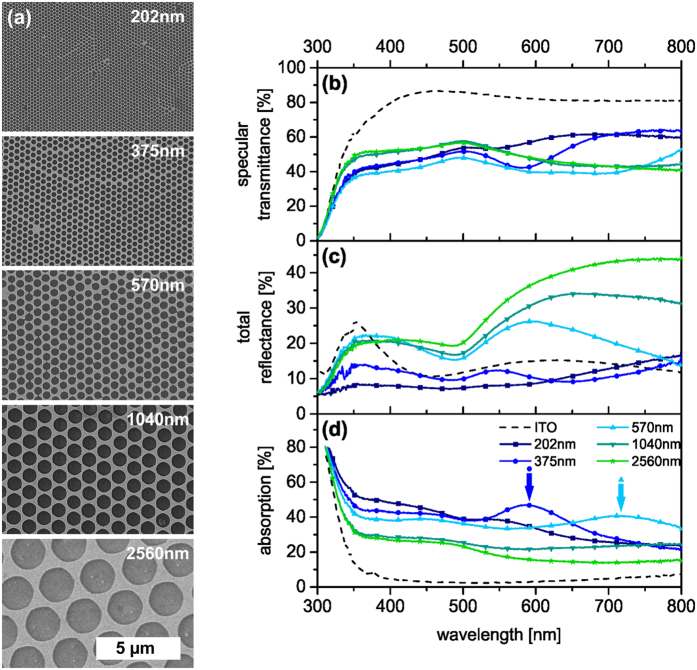
Electron microscopy and optical characterization of gold electrodes with constant gold area fraction. The corresponding hole diameters are listed in Table 1. (**a**) SEM images of gold nanohole arrays with different periodicities. The numbers given in (**a**) indicate the corresponding periodicity *P*. (**b**) Specular transmittance, (**c**) total reflectance and (**d**) absorption spectra of ITO (dashed line) and nanohole arrays (solid lines). The arrows indicate the positions of the SPP resonances.

We fabricated our large area nanohole arrays via colloidal lithography with a metal layer thickness of 50 nm^41^,^42^,^43^,^44^. We used non-close-packed polystyrene particle monolayers prepared by self-assembly at the liquid/air interface and subsequent dry etching of the particles^42^. These monolayers then act as templates for the deposition of a gold layer by thermal evaporation. Finally, the particle template is removed yielding the hexagonally ordered nanohole array. In this periodic arrangement, the localized excitation of the surface plasmon resonance of the metallic nanoholes interferes with the far-field from the Bragg diffraction mode of the lattice (Rayleigh anomaly) leading to a Fano-type surface lattice resonance with narrow and asymmetric line shape^45^,^46^. The spectral position of the SPP resonances thereby depends on the predefined periodicity (center-to-center distance between the holes *P*^17^). *P* is given by the initial diameter of the polystyrene particles. Using polystyrene particles with diameters of 202 nm, 375 nm, 570 nm, 1040 nm and 2560 nm we were able to cover the optical range from visible to near-infrared. SEM images of the resulting gold patterns are shown in Fig. 1a. The diameter of the holes *d* was adjusted to a constant *d/P* value of approximately 0.8 to obtain a constant area fraction. Consequently, around 40% of the device area, independent of the periodicity, was covered by gold. The sheet resistance for Au was in the range of 8–18 Ω/□ for this surface coverage^37^. Further increasing the diameter of the holes would result in higher sheet resistances and might cause a reduction in the charge extraction in the solar cell devices due to the limited diffusion length of the charge carriers^37^. Since the sheet resistance of the nanomesh electrodes is comparable to standard ITO substrates, differences in the device performance can be solely attributed to the optical and plasmonic properties of these electrodes. Figure 1b and c depict the optical properties of the uncoated nanohole arrays with a systematic variation in periodicity on a glass substrate.

For all periodicities, the transmittance ranges between 40–60% in a wavelength range of 350 nm to 800 nm and is therefore significantly lower compared to the reference ITO electrode which has more than 80% transmittance. (Fig. 1b). However, the transmittance data only provide limited information for tailoring the light management in a solar cell device. In particular, for plasmonic structures the discrimination between scattering, reflection and absorption is essential. Thus, the optical measurements were performed in an integrating sphere with the light incident onto the gold structures through the glass substrate. The wavelength dependent contribution of the total reflectance and absorption are shown in Fig. 1c and d, respectively. At wavelengths below 370 nm, all nanohole arrays are less reflective than ITO, due to the different band transitions of gold and ITO. Yet, their strong absorption at higher wavelengths results in an overall lower transmittance.

At the same time one can clearly discern periodicity-dependent changes of the optical properties. The nanohole arrays with *P* = 202 nm and *P* = 375 nm exhibit a lower reflectance compared to ITO across the whole range of interest. With increasing periodicity the reflectance increases, whereas the absorption decreases. This behavior is reminiscent of isolated plasmonic particles, which also show a size-dependence of their scattering to absorption ratio^24^. Additionally, these overall trends are superimposed by characteristic dips in the transmittance spectra. These originate from the excitation of SPP modes and are based on dipolar mode oscillations (simulated spectra of the pure nanomeshes and electric field distributions at the respective resonance wavelengths are given in Supplementary Figs S1 and S2). These transmittance minima correspond to slightly redshifted maxima in the absorption spectra (highlighted in Fig. 1d) and thus show an asymmetric signature of the Fano resonance line shape. The SPP resonance strongly shifts to higher wavelength with increasing periodicities. Hence, in the relevant wavelength range for organic solar cells studied here (300 nm–800 nm), the plasmonic resonances are visible only for the periods *P* = 375 nm at 585 nm and for *P* = 570 nm at 715 nm. Moreover, for the smallest period (*P* = 202 nm) no resonance is discernible due to high losses in the gold near the intraband transition.

The fundamental question we want to address is, whether the expectedly high optical losses caused by the lower transmittance compared to the ITO reference, can be compensated by potential plasmonic enhancement effects. From a solar cell light management point of view, the resonance wavelength of the SPP mode should overlap with the absorption of the active medium. In that case, the enhancement of the electric field due to the resonance may result in additional absorption in the photoactive material. In a functional photovoltaic device, the light management will be further complicated by the interaction of the SPP mode with the metallic back electrode. This is known to result in an additional mode known as mirror charged mode, cavity mode (metal-active layer-metal) or magnetic mode^47^,^48^,^49^.

**Figure 2:**
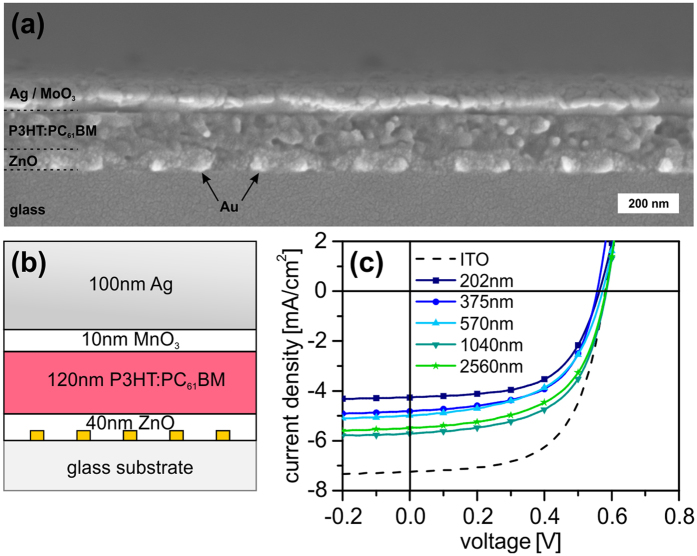
P3HT: PC_61_BM solar cells based on gold nanohole arrays. (**a**) SEM cross-section of a P3HT:PC_61_BM device with *P* = 202 nm (a cross-section measured with the backscattered electron (BSE) detector is given in Supplementary Fig. S5). (**b**) Schematic illustration of the inverted device structure with a gold nanohole electrode. (**c**) Current-density – voltage characteristics under illumination of solar cells built on ITO (dashed) and gold nanohole arrays with different periodicities.

Solar cells were fabricated using two different (P3HT:PC_61_BM and PTB7:PC_71_BM photoactive layers) on top of nanomesh electrodes introduced above. The exact inverted cell layout is given in Fig. 2a and b for the nanomesh devices and in Supplementary Fig. S3 for the ITO reference device. Figure 2c shows the J-V curves under illumination of the P3HT:PC_61_BM devices for all periodicities (dark current characteristics are shown in Supplementary Fig. S4). The solar cell performance for each case was checked for reproducibility and consistency by repeating the experiment on different days. Table 1 summarizes the best and averaged (in parenthesis) solar cell performance parameters obtained for each case. The reference solar cell devices on an ITO electrode showed a power conversion efficiency (PCE) of 2.6% for the P3HT:PC_61_BM photoactive layer. In general, all nanohole array based solar cells showed a reduced PCE in comparison to the reference solar cell.

Compared to the ITO reference the J_SC_ dropped from 6.8 mA/cm^2^ to 4.2 mA/cm^2^ for the smallest periodicity. Nevertheless, the J_SC_ and consequently the PCE increased monotonically with *P* up to a maximum for 1040 nm followed by a decrease at 2560 nm. The maximum PCE for the P3HT:PC_61_BM active layer with nanomesh electrode (*P* = 1040 nm) was 2.0%, which is ~77% of the ITO reference device. It should also be noted that within each case, the open circuit voltage (V_oc_) and the fill factor (FF) values remained nearly constant. This suggests the absence of excessive leakage currents and that the ZnO layer fully covered the nanomeshes. Besides that, the observed high FF value also indicates that the metal nanohole array was homogeneously closed. The nanomeshes offered a comparable electrical conductivity relative to the conventional ITO electrode. Finally, even for the largest apertures (~2100 nm), the solar cells are not restricted by the charge carrier diffusion length in the ZnO blocking layer. Thus, we conclude that the nanomesh based solar cells were not electrically limited and that any deviations from the ITO reference were optically driven.

**Figure 3:**
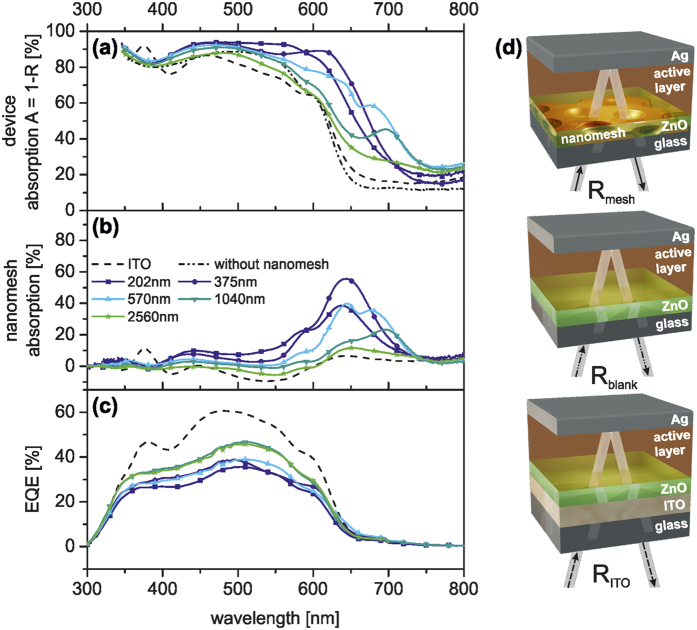
Influence of nanohole arrays on absorption and photocurrent generation in P3HT:PC_61_BM solar cells. (**a**) Absorption spectra of P3HT:PC_61_BM solar cells with gold nanohole electrodes with different periodicities (solid lines), of the same solar cell without the nanomesh (dash-dotted line), and of the P3HT:PC_61_BM reference device on ITO (dashed line). (**b**) Contribution of the nanohole electrode to the device absorption. The nanomesh absorption is obtained by subtracting the absorption (A = 1 − R) of the same solar cell device without a nanomesh electrode (R_blank_, dash-dotted line, Fig. 3d) from the device absorption with nanomesh electrode (R_mesh_, solid line). (**c**) EQE spectra of the P3HT:PC_61_BM solar cells with nanomesh electrodes (solid lines) compared to the ITO reference cell (dashed line). (**d**) Schematic presentation of the solar cell devices used for the reflectance measurements with (top) and without (center) the nanohole array electrode and with the ITO electrode (bottom).

Therefore, changes in J_SC_ can be rationalized by the optical properties of the fully assembled solar cell devices (Fig. 3). The absorption (A) of the solar cells was calculated from the total reflectance (R) measurements via A = 1 − R. In the range from 400 nm to 750 nm, the overall device absorption (Fig. 3a) is significantly higher than that of the reference device with ITO (dashed line). The smaller the period *P*, the greater the absorption compared to the reference device, which is in good accordance with the absorption measurements of the pure nanomeshes (Fig. 1d). Furthermore, an additional absorption peak appears close to the absorption edge of P3HT (600 nm–650 nm) for the nanomesh based devices, which is not visible for the ITO reference device. This peak shifts red from 625 nm to 700 nm for increasing lattice periods. It is most pronounced for *P* = 1040 nm and disappears again for *P* = 2560 nm. We attribute this additional absorption to light trapping caused by the electrode grating.

To single out the contribution of the nanomesh electrodes to the device absorption we calculated the nanomesh absorption. This was done by subtracting the absorption of the device stack without the nanomesh electrode (A_blank_ = 1 − R_blank_) from the absorption of the device stack with the nanohole array electrode (A_mesh_ = 1 − R_mesh_) and is displayed in Fig. 3b and d. This is the most insightful optical characterization as it provides a direct measure of the absorption within the device (based on the absorption properties of the photoactive layer and the reflective back electrode) and the contribution of the nanohole electrode. As surface plasmons are highly sensitive towards the refractive index environment, the absorption of nanohole arrays in the device differs from the absorption of the neat metal arrays. Thus, a correlation between the device performance and the optical properties of the pure meshes is not always directly possible. One can infer quite clearly that the absorption of the device is mainly dictated by the photoactive layer. From 300 nm to 550 nm only a slight broadband offset in the absorption can be assigned to the nanohole array. However, approaching the absorption band-edge of the photoactive polymer a strong increase of the entire device absorption can be seen for all but the largest periodicity *P* = 2560 nm. The maximum of the nanomesh contribution to the absorption was found for *P* = 375 nm. However, this additional absorption does not contribute to the device performance as inferred from the external quantum efficiency (EQE). Figure 3c shows EQE only in the absorption range of the photoactive layer. Since no enhancement of the EQE in the wavelength range from 550 nm to 750 nm is observed, it can be concluded that the high absorption within the nanohole arrays does not lead to photon to electron conversion. Moreover, the EQE is reduced for nanomesh based solar cells in the whole visible range compared to the ITO reference device. The smaller the grating period and thus the larger the additional absorption due to the nanomesh, the lower the EQE of the device. Therefore, the increasing PCE for larger periodicities can rather be assigned to the decrease of the parasitic absorption losses, which are in good agreement with Fig. 1d. Additionally, for the largest periodicity (*P* = 2560 nm) the reflectance losses at the nanomesh front electrode result in a decreased device absorption. Thus, a periodicity of *P* = 1040 nm presents the best trade-off to minimize both the parasitic absorptive and the reflectance losses.

**Figure 4:**
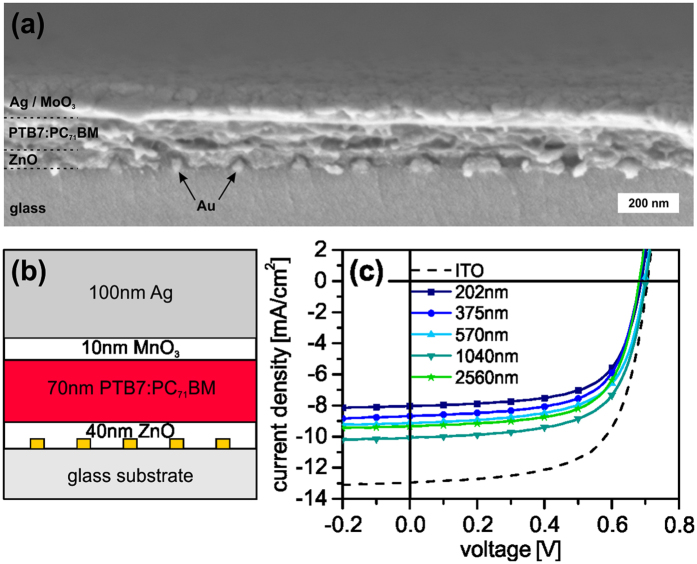
Device characteristics of PTB7:PC_71_BM solar cells based on gold nanohole arrays. (**a**) SEM cross-section of a PTB7:PC_71_BM device with *P* = 202 nm. (**b**) Schematic illustration of the inverted device structure with a gold nanohole electrode. (**c**) Current-density – voltage characteristics under illumination of solar cells built on ITO (dashed line) and gold nanohole array TCEs with different periodicities.

In order to capitalize on the increased absorption between 600 nm and 700 nm due to the nanohole electrode, we prepared solar cell devices based on a low band-gap polymer PTB7 and the fullerene derivative PC_71_BM. The PTB7:PC_71_BM blend absorbs up to a wavelength of 750 nm, thus covering the wavelength range of the maximum nanomesh absorption in Fig. 3b. The inverted device structure and the J-V curves of the PTB7:PC_71_BM devices are shown in Fig. 4.

**Figure 5:**
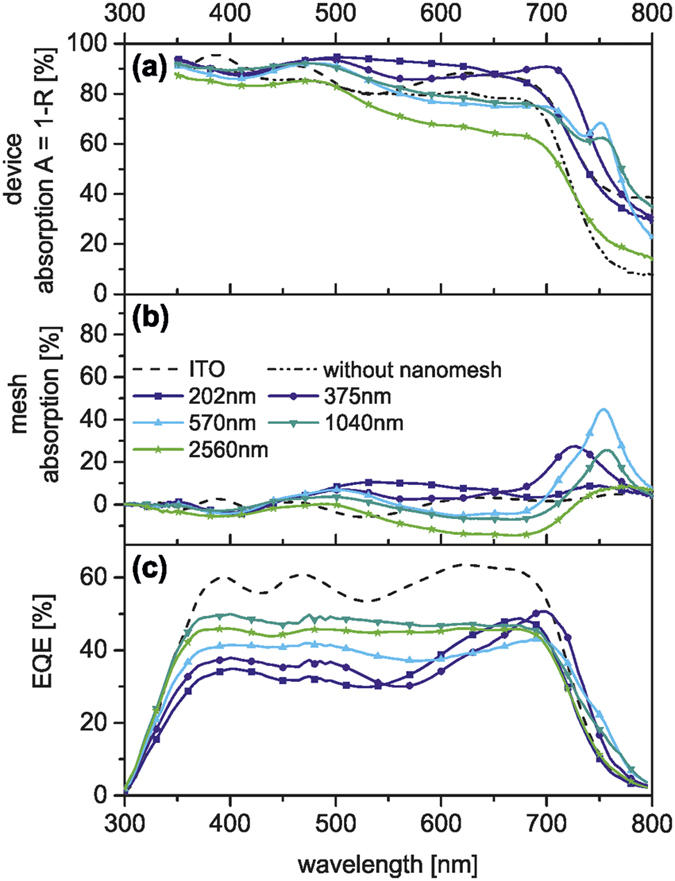
Influence of nanohole array on absorption and photocurrent generation in PTB7:PC_71_BM solar cells. (**a**) Absorption spectra of PTB7:PC_71_BM solar cells with gold nanohole electrodes for different periodicities (solid lines), of the same solar cell without the nanomesh (dash-dotted line), and of the PTB7:PC_71_BM reference device on ITO (dashed line). (**b**) Contribution of the nanohole electrode to the device absorption. The nanomesh absorption is obtained by subtracting the absorption (A = 1-R) of the same solar cell device without a nanomesh electrode (R_blank_, dash-dotted line) from the device absorption with nanomesh electrode (R_mesh_, solid line, compare Fig. 3d). (**c**) EQE spectra of the PTB7:PC_71_BM solar cells.

A similar trend compared to the P3HT:PC_61_BM solar cells was observed for the solar cell parameters of the PTB7:PC_71_BM devices with the various periodicities of the nanomesh TCE (Table 2). The J_SC_ drops from 12.8 mA/cm^2^ to 7.7 mA/cm^2^ for the smallest periodicity. Increasing *P* monotonically increases the J_SC_, which again decreases beyond *P* = 1040 nm. The maximum PCE was 4.6% for *P* = 1040 nm compared to 6.0% for the reference solar cell devices based on ITO. Again, no excessive leakage currents were observed for the nanohole devices and the open circuit voltage (V_OC_) and the fill factor (FF) values were nearly constant for all periodicities. The corresponding device absorption is given in Fig. 5a.

Compared to the P3HT:PC_61_BM devices, a similar trend in the device absorption was obtained for the PTB7:PC_71_BM devices. The absorption steadily decreases with increasing lattice periodicity. This can be attributed to the decreasing plasmonic absorption and increasing reflectance of the nanohole arrays. In contrast to P3HT:PC_61_BM, the absorption of the PTB7:PC_71_BM nanomesh devices hardly exceeds the absorption of the ITO reference device in the range up to 700 nm, where the active layer is strongly absorbing. Instead, for large periodicities the total absorption decreases compared to the ITO reference device. Again, the overall device absorption profile is strongly dictated by the absorption properties of the photoactive polymer, which extends up to the absorption edge (750 nm) of PTB7:PC_71_BM blend. Surprisingly, the additional absorption peaks evoked by the plasmonic properties of the nanohole arrays are now strongly redshifted compared to those in the P3HT:PC_61_BM systems. For *P* = 375 nm, *P* = 570 nm and *P* = 1040 nm additional absorption peaks are visible at the absorption edge of the polymer between 700 nm and 750 nm. We note that the highest nanomesh absorption is now observed for the *P* = 570 nm electrode instead of the *P* = 375 nm for the P3HT:PC_61_BM blends (Fig. 5b). Interestingly, the absorption of the nanohole electrode with *P* = 202 nm incorporated in the PTB7:PC_71_BM device stack exhibits a spectrally flat behavior similar to ITO, while a distinct absorption peak is visible for the same structure in the P3HT:PC_61_BM device. Again, the absorption related to the plasmonic electrode is absent for the largest grating period (*P* = 2560 nm).

Similar to the P3HT:PC_61_BM devices, the EQE of the PTB7:PC_71_BM devices is also reduced compared to the ITO reference device. However, among the nanomesh devices the EQE increases in a broad range from 350 nm to 650 nm for larger periodicities due to a decrease of the parasitic absorption losses (Fig. 5c). Moreover, for PTB7:PC_71_BM the EQE of the devices with *P* = 202 nm and *P* = 375 nm exhibit asymmetric line shapes with a strong increase in the EQE above 550 nm, whereas larger grating periods yield rather flat EQE spectra. For wavelengths above 720 nm, in a range where the absorption of the PTB7:PC_71_BM blend is already strongly reduced, the EQE of the nanohole devices even surpasses the EQE of the ITO reference device.

This EQE enhancement spectrally coincides with the absorption peaks assigned to the plasmonic resonances and redshifts with increasing periodicity of the nanomesh electrode. Thus, we attribute this increase in EQE above 550 nm to the light trapping behavior of the plasmonic electrode. Consequently, the plasmonic resonances induced by nanohole arrays indeed contribute to the photocurrent of the device. Therefore, we can claim that plasmonic absorption enhancement is possible via these periodically structured electrodes. However, the gain in EQE is not sufficient to outweigh the inevitable transmission losses in the range from 350 nm–700 nm, where the polymer is highly absorbing. Compared to the ITO device, the EQE spectra of the nanohole devices are only enhanced above 720 nm, where the absorption of the polymer is the limiting factor for the device efficiency. For the largest periodicity *P* = 2560 nm, no enhancement in the EQE can be observed presumably due to its predominantly reflecting behavior at the wavelength range of interest. This trend strongly indicates that for large lattice periodicities, the plasmonic influence is negligible and the electrodes have to be optimized for their overall transmittance in the first place.

The comparison between the P3HT:PC_61_BM and PTB7:PC_71_BM devices reveals an intriguing dilemma when trying to make use of plasmonic enhancement and replacing the ITO electrode simultaneously. Whereas the resonance wavelength of the pure meshes is strongly dependent on the grating periodicity, the overall device absorption is more strongly determined by the absorption of the photoactive layer. The previously broadband SPP resonances become strongly confined to the narrow spectral range, which is defined by the absorption edge of the active layer material. Thus, although the same electrode periodicities have been used for both blend systems, the high plasmonic absorption between 600 nm and 650 nm of the P3HT:PC_61_BM devices was redshifted to the band-edge of PTB7:PC_71_BM devices (700 nm–750 nm), where the exciton conversion is again less efficient. Even worse, the optimum coupling was observed for *P* = 375 nm and *P* = 570 nm. Yet, the optimum PCE is found for *P* = 1040 nm. This demonstrates even more drastically that losses in the transmittance cannot be counteracted by a plasmonic enhancement.

**Figure 6:**
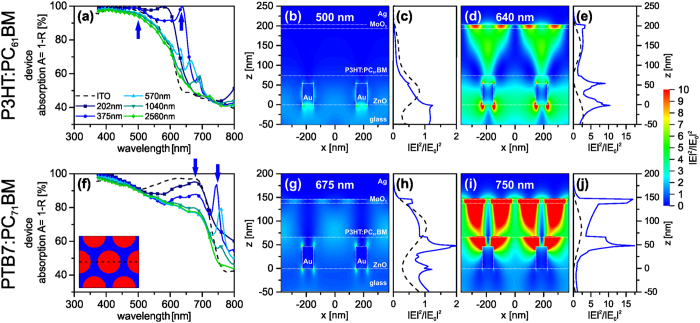
FDTD simulation of the P3HT:PC_61_BM (top row, a–e) and PTB7:PC_71_BM (bottom row, f–j) solar cell devices. Simulated spectra of (**a**) P3HT:PC_61_BM and (**f**) PTB7:PC_71_BM solar cells with gold nanohole electrodes and different periodicities are compared to those of ITO reference devices. The arrows indicate the two characteristic incident wavelengths used for the simulation of the electric field enhancement as shown in (**b**,**d**,**g** and **i**). Electric field intensity |E|^2^ distributions normalized to the incident electric field intensity |E_0_|^2^ for P3HT:PC_61_BM at 500 nm (**b**) and 640 nm (**d**) and for PTB7:PC_71_BM at 675 nm (**g**) and 750 nm (**i**). The electric field enhancement shown in (**b**,**d**,**g** and **i**) were evaluated along the cross-section (dashed line) in the inset of (**f**) for *P* = 375 nm in all cases. In (**c**,**e**,**h** and **j**) electric field enhancement averaged over the xy-plane of the unit cell is depicted along the z-coordinate for the respective wavelength for the ITO device (dashed line) and the nanomesh device (solid line).

To understand the impact of the nanomesh optics on the two different photoactive material systems in more detail, the electric field distributions of the plasmonic modes were simulated by the finite-difference time-domain (FDTD) method (Fig. 6). The simulated device absorption spectra for P3HT:PC_61_BM (Fig. 6a) and PTB7:PC_71_BM (Fig. 6f) qualitatively match the experimental trend (for transmittance and reflectance spectra see Supplementary Figs S6 and S7). Especially, the experimentally observed redshift of plasmonic absorption from P3HT:PC_61_BM to the PTB7:PC_71_BM is clearly supported by the simulation curves. In the region of strong photoactive layer absorption, the device absorption decreases with increasing nanohole periodicities. At the absorption edge pronounced absorption peaks are visible, which redshift with *P* and become less intense. The electric field distribution was simulated in three dimensions and plotted along the cross-section (dashed line) shown in the inset of Fig. 6f for both active layer materials at two different characteristic wavelengths (bold arrows in Fig. 6a and f) for a nanohole electrode with *P* = 375 nm. The enhancement of the electric field intensity |E|^2^ in the device structure compared to the incident light intensity |E_0_|^2^ is depicted for the P3HT:PC_61_BM devices in Fig. 6b and d and for the PTB7:PC_71_BM devices in Fig. 6g and i. In Fig. 6c,e,h and j the corresponding electric field profiles |E|^2^/|E_0_|^2^ averaged over the whole unit cell along the layer stack direction are given for the nanomesh devices (solid lines) compared to the ITO reference devices (dashed lines). The electric field distributions for the ITO reference devices are depicted in Supplementary Fig. S8.

At first, we want to assess the field distribution at a wavelength, which is lower than the absorption edge and dominated by the active medium absorption. Therefore, the field distribution was calculated at 500 nm (Fig. 6b) and 675 nm (Fig. 6g) for P3HT:PC_61_BM and PTB7:PC_71_BM, respectively. In each case, a nanohole array with *P* = 375 nm was simulated, since this periodicity demonstrated a strong contribution of the nanohole absorption in either case (Figs 3a and 5a). At these wavelengths, the corresponding photoactive layers absorb very strongly, and the solar cells are mainly limited by parasitic absorption and reflectance losses. For P3HT:PC_61_BM (Fig. 6b) the simulation shows the scattering of the incoming light at the nanomesh with a close confinement of the electric field to the edges of the gold structure and a field profile, which exponentially decays into the active layer. The nanomesh is strongly reflecting resulting in high normalized field intensities exceeding unity in the glass layer (Fig. 6c). In general, owing to the high attenuation of the photoactive layer, the electric field intensity in the layer stack is mainly reduced compared to the incident light intensity. Moreover, the electric field intensity in the photoactive layer is reduced for the nanomesh device relative to the ITO reference device. This gives rise to a decreased absorption in the nanomesh device at this wavelength. For the PTB7:PC_71_BM active layer at 675 nm (Fig. 6g) a dipolar plasmon resonance is excited, which is weakly coupling to the silver back electrode. This can be inferred from the field enhancement in the photoactive layer and the MoO_3_ layer (Fig. 6h). Nevertheless, the electric field intensity in the photoactive layer of the nanomesh device does not surpass the ITO reference device, which features a maximum of the field profile in the photoactive layer. Consequently, the contribution of the plasmonic resonance to the photocurrent of the device is negligible. This corroborates the experimental finding that the optical properties of the device are predominantly governed by the photoactive layer. A high absorption in the photoactive layer attenuates the coupling between the nanomeshes and the back electrode and consequently it inhibits the evolution of confined plasmonic modes in the wavelength region of interest. However, to get some considerable improvements, these confined plasmonic modes are required to efficiently generate a surplus of excitons to outweigh the losses originating from the reduced transmittance through the nanohole electrode.

Field distribution simulation was also carried out near the absorption edge of the respective photoactive systems: at 640 nm and 750 nm for P3HT:PC_61_BM and PTB7:PC_71_BM, respectively. Contrary to the field distribution observed within the absorption range of the photoactive layer discussed above, in the region of the absorption edge of the respective photoactive layers the electric field is less confined to the nanomesh electrode. Instead, the electric field intensity within the active layer of the devices is enhanced with respect to the incident intensity. For P3HT:PC_61_BM the electric field in the photoactive layer is moderately enhanced by a factor of 1.2 when excited at 640 nm, attributed to the resonance of the gold structure. Mirror charges are induced at the back electrode with high field intensities occurring in the MoO_3_ layer near the Ag surface (Fig. 6e). Symmetrically enhanced electric fields at the edges of the gold structure indicate a strong coupling of the incident light with the plasmonic resonance. The localization of the electric field inside the active layer clearly indicates the presence of a cavity mode at this wavelength^19^. Inside the photoactive layer of the nanomesh device the electric field intensity even exceeds the intensity observed in the photoactive layer of the ITO reference device. Thus the light entrapment at this wavelength could indeed contribute to a plasmonically driven enhanced exciton generation.

An even stronger electric field enhancement was found for the PTB7:PC_71_BM device around the absorption maximum of the metal electrode at 750 nm in Fig. 6i, showing an enhancement factor of up to 15 in the MoO_3_ layer and 5.6 averaged over the photoactive layer. We assign this higher electric field enhancement to the smaller distance (70 nm) between the two metallic electrodes in the PTB7:PC_71_BM devices compared to that (120 nm) in the P3HT:PC_61_BM devices. The plasmonic resonance emerging from the nanohole electrode is enhanced by the presence of the Ag back electrode, which depends on the length of the cavity between the electrodes^19^,^50^. For thinner devices the coupling between the structured front and the flat back electrode is expected to be stronger. Although the 70 nm photoactive layer thickness used here may not be the optimum thickness to attain a maximum EQE enhancement at the absorption edge, even an optimized active layer thickness would not prevent the reflectance and absorption losses of the gold structure at the more relevant lower wavelengths (300–600 nm).

The low field intensity at the gold/glass interface (Fig. 6j) points towards a low reflectance and absorption of the gold nanomesh. Here the electrode predominantly couples the incident light forward into the photoactive layer. Consequently, the losses caused by the gold nanomesh are minimal at this wavelength leading to the desired light concentration in the photoactive layer. Whereas PTB7 is only weakly absorbing at 750 nm, the plasmonic field enhancement still leads to a higher exciton generation in the active layer compared to the ITO device. This manifests itself in a higher EQE as shown in Fig. 5c. At an even higher wavelength of 800 nm, which is off-resonance, one can still observe a field enhancement in the active layer (Supplementary Fig. S9). However, it is more confined to the ZnO rather than the active layer. In combination with the low polymer absorption at this wavelength no additional exciton generation and thus no contribution to the photocurrent in the EQE spectrum is seen. Simulated field distribution maps at the absorption maximum of all other periodicities for both photoactive layers also demonstrate the evolution of a cavity mode (see Supplementary Figs S10 and S11). While this can be excited with a broad range of periodicities, the coupling strength decreases with increasing *P*.

The discrepancy between losses in the short wavelength region and EQE enhancement at long wavelengths has already been described for Au nanowire electrodes^7^ and nanoparticle arrays placed on the ITO electrode^51^. While the particles reduced the efficiency at small wavelength due to absorption and reflectance losses, a slightly enhanced efficiency was found at the absorption edge of the active layer attributed to a diffractive scattering or collective plasmonic mode. This scattering mode can only exist at the absorption edge of the active layer as the electric field from the particles would be suppressed by a higher attenuation of the polymer^52^. Instead, nanostructured back contacts result in an EQE enhancement pinned to the absorption edge of the polymer, but naturally without transmittance losses^26^,^53^,^54^,^55^,^56^.

To further clarify the nature of the cavity resonance, additional FDTD simulations were conducted in which we substituted the gold of a nanomesh with *P* = 375 nm by ITO (Supplementary Fig. S12). ITO does not show any plasmonic resonances in the wavelength range of interest and is purely scattering the incident light due to the periodic nanostructure. Thus, the absence of the absorption maximum at 750 nm clearly indicates that this resonance is of plasmonic origin.

## Conclusion

In summary, we systematically investigated the chances and limitations of thin plasmonic nanomeshes as transparent conducting electrodes for polymer solar cells. We, therefore, prepared gold nanohole arrays by colloidal lithography covering periodicities from 202 nm up to 2560 nm. These plasmonic nanohole arrays support SPP modes, which are governed by the underlying periodicity, and cover a broad optical range.

The nanohole electrodes were incorporated into organic solar cells consisting of two different photoactive layer polymer blends, the standard material P3HT:PC_61_BM and the low band-gap material PTB7:PC_71_BM. No losses in the V_OC_ and FF of the devices indicated the absence of excessive leakage currents and high conductivities of the metal meshes. This renders such electrodes electrically comparable to the ITO reference. For both photoactive polymers, we found appreciable PCEs in our ITO-free devices reaching up to 77% of the reference solar cells. This decreased PCE was not surprising based on the strongly reduced transmittance of the nanohole electrode. Interestingly, the PCE increased with the grating period of the nanomeshes. An optimum was found in both polymer blend cases for *P* = 1040 nm, which was attributed to an optimum balance between low losses by parasitic absorption and low losses by increasing the reflectivity of the nanomesh.

Plasmonic field enhancement due to the excitation of SPP and cavity modes within the solar cell stack was expected to be able to counteract these losses. We indeed did observe additional absorption peaks in the spectra of the nanomesh devices but only at the absorption edge of the respective polymer. Using FDTD simulation we were able to assign these to the excitation of a cavity mode. However, since this enhanced mode only exists at or below the absorption edge of the particular polymer, no enhancement of the photocurrent was observable for P3HT:PC_61_BM. For PTB7:PC_71_BM the cavity mode led to an EQE enhancement beyond 720 nm, however, this additional contribution was marginal compared to parasitic absorption and reflection losses at lower wavelengths. Consequently, surface plasmon resonances play an ambivalent role in the device optimization of polymer solar cells. They are capable of improving the absorption within a small spectral region. However, the broadband losses at any other wavelength cannot be compensated. To replace ITO without any losses in PCE, traditional methods to increase the overall transmittance such as decreasing the metal layer thickness or reducing the metal surface coverage are expected to have a greater impact (as long as the electrical properties are not compromised). Nevertheless, we want to conclude that this approach is still capable of producing ITO-free solar cells with reasonable PCE’s based on standard photoactive material systems.

## Methods

### Materials

Polystyrene particles were purchased from *Microparticles GmbH* (Berlin) or synthesized using emulsifier-free emulsion polymerization. P3HT (Rieke Metals – 4002-EE), PTB7 (Solarmer – ZP002), PC_61_BM (Solenne) and PC_71_BM (American Dye Source) were obtained from commercial suppliers and used without any further purification. The thickness and the sheet resistivity of our reference ITO substrates were 213 nm and 10 Ω/□, respectively.

### Fabrication of Au nanomeshes

Monolayers of polystyrene particles were prepared according to the procedure of Retsch *et al*.^42^ Cationically functionalized glass slides were spin cast with a 3 wt% particle dispersion at a speed of 4000 rpm. Subsequently, the coated glass substrates were immersed in a 0.1 mM SDS solution in MilliQ. The aqueous phase was adjusted to pH 12 by adding a few drops of NH_3_. A monolayer was formed at the liquid/air interface by self-assembly of the detaching particles. The monolayer was transferred to a 1 × 1 inch glass substrate and dried in air. The monolayers were etched in a plasma reactor MiniFlecto (*Plasma Technology GmbH*, Herrenberg, Germany) with 75% argon and 25% oxygen at 80 W at a pressure of 0.14 mbar to obtain non-close packed monolayers. A 3 nm chromium layer and 50 nm gold were deposited using a Balzers BA360 thermal evaporation chamber. The layer thickness was monitored via a SQM 160 microbalance (*Sigma Instruments, Schaefer Technologie GmbH*). Afterwards, the particles were removed using Scotch® tape (3M) giving the nanohole arrays. The Au substrates were cleaned for 10 min in an ultrasonic bath with a 2% aqueous Hellmanex (*Hellma GmbH*, Mühlheim, Germany) solution in MilliQ water. The surfactant was extensively rinsed off with MilliQ water, and the substrates were placed in the ultrasonic bath in ethanol for 10 minutes and dried with compressed air.

### Solar cell fabrication

Solar cell devices were prepared by spin coating a zinc acetate solution (109.75 mg zinc acetate dehydrate, 30.5 μL ethanol amine and 1 ml methoxyethanol) onto cleaned substrates with patterned nanomeshes, followed by 150 °C baking for 5 min in air to convert zinc acetate to zinc oxide. The reference devices were on ITO glass substrates. The film thickness of the ZnO films was around 40 nm. Subsequently, the substrates were transferred to a glovebox for the deposition of the photoactive layer in a nitrogen environment. P3HT:PC_61_BM films (~115 nm) were prepared by spin-coating (850 rpm) 80 μl of a solution containing 16.8 mg of P3HT and 13.2 mg of PC_61_BM in 1 ml of chlorobenzene. Subsequently, the P3HT:PC_61_BM films were annealed at 135 °C for 15 min. PTB7:PC_71_BM films (~80 nm) were prepared by spin-coating (1000 rpm) 90 µl of a solution containing 12 mg PTB7, 19.2 mg PC_71_BM and 50 µl of 1,8-diiodooctane (DIO) in 1 ml of o-xylene. The films were dried in a glovebox anti-chamber for 30 min to remove residual DIO. The respective active layer thicknesses were adjusted to the optimum charge carrier mobility in the devices. Top electrodes consisting of MoO_3_ (10 nm) capped by Ag (150 nm) were deposited by vacuum evaporation at ~1 × 10^−6^ mbar.

### Characterization

I-V measurements were performed under an inert environment with a Keithley 2400 source measure unit under 100 mW/cm^2^ illumination from an AM 1.5 class A solar simulator. The active area of 9 mm^2^ was defined by the overlap of a black mask aperture area, the ITO or nanomesh electrode, and the evaporated top electrode. External quantum efficiency (EQE) measurements were performed under both dark and white light bias conditions at short circuit conditions via a Bentham PVE 300 assembly unit. More details have been published elsewhere^57^.

UV/VIS spectra were measured using a Cary 5000 UV-Vis-NIR Spectrophotometer (*Agilent Technologies*) with attached Diffuse Reflectance Accessory between 300 and 1200 nm at an angle of incidence of 8° with an UV-bandgap of 3 nm, an IR-bandgap of 12 nm, a data interval of 1 nm, and a scan speed of 600 nm/min.

The sheet resistance of the nanomeshes was measured with a Lucas Signatone SYS-301 and a SP4 probe head.

SEM images were taken on an LEO 1530 Gemini Field Emission SEM (*Carl Zeiss AG*, Oberkochen, Germany) and an Ultra plus Field Emission SEM (*Carl Zeiss AG*, Oberkochen, Germany). The images were evaluated with the software ImageJ^58^.

### FDTD Simulation

To determine the refractive index of the glass substrate, ZnO, MoO_3_, P3HT:PC_61_BM and PTB7:PC_71_BM we used an M2000 spectroscopic ellipsometer from *J. A. Woollam Co.* in the wavelength range from 245 nm to 1600 nm (D2 and QTH lamps). Ellipsometric data from all samples were acquired at five different angles of incidence in five-degree steps. To determine the refractive index of all materials we used a general oscillator layer model within the CompleteEASE (Version 5.07) software. All modeling approximation were physical reasonable (parametrization to fulfill Kramers–Kronig relations) and showed a mean square error (MSE) below five.

The optical response in transmission and reflection at normal incidence and unpolarized light was simulated using a commercial software from *Lumerical Solutions, Inc.* (FDTD Solutions, Version 8.11.422). We used a hexagonal hole structure with periodic boundary condition (BC) in later direction and perfect match layer (PML) BC in beam direction with a linear polarized plane wave source. Hole diameters, periodicity, the amount of multilayer materials and thickness were obtained from the experiment. In beam direction, the FDTD simulation total length was chosen to be 4 μm with transmission monitors located at both ends. The simulation setup has been placed in the center of the FDTD simulations, and the plane wave source was injected starting from the glass layer. For a broadband source simulation (λ = 300–800 nm), the FDTD software approximates the refractive index of the materials by a polynomial function (the refractive indices determined via spectral ellipsometry and the FDTD approximations are given in Supplementary Figs S13–S17). All optical constants were approximated with an RMS error below 0.21. An anisotropic mesh overwrite region were used according to the specific periodicity and hole diameter (mesh in lateral direction: between 2 nm mesh for *P* = 202 nm and 6 nm mesh for *P* = 2560 nm, mesh in beam direction: always 2 nm). All simulations reached the auto shut-off level of 10^−5^ before reaching 1000 fs simulation time. To determine the electric field distribution and surface charge densities, we simulated the model at the plasmonic mode frequency at a pulse length of ~20 fs (optimized for long pulse length).

## Additional Information

**How to cite this article:** Stelling, C. *et al*. Plasmonic nanomeshes: their ambivalent role as transparent electrodes in organic solar cells. *Sci. Rep.* **7**, 42530; doi: 10.1038/srep42530 (2017).

**Publisher's note:** Springer Nature remains neutral with regard to jurisdictional claims in published maps and institutional affiliations.

## Supplementary Material

Supplementary Information

## Figures and Tables

**Table 1:** Optimum and average of five (in parenthesis) P3HT:PC_61_BM device parameters.

*P* [nm]	*d* [nm]	AF_Au_ [%]	R_sh_ [Ω/□]	J_SC_ [mA/cm^2^]	V_OC_ [V]	FF [%]	PCE [%]
ITO reference	—	—	17.4	7.3 (6.8)	0.58 (0.59)	61 (62)	2.6 (2.5)
202	155	46	15.7	4.3 (4.2)	0.56 (0.56)	60 (60)	1.4 (1.4)
375	293	46	10.0	4.6 (4.6)	0.57 (0.56)	58 (53)	1.5 (1.4)
570	462	43	8.5	5.1 (5.1)	0.57 (0.56)	55 (53)	1.6 (1.5)
1040	853	37	17.3	5.7 (5.7)	0.59 (0.58)	58 (60)	2.0 (2.0)
2560	2077	37	8.5	5.4 (5.4)	0.58 (0.58)	58 (58)	1.8 (1.8)

*P*: nanomesh periodicity, *d*: hole diameter, AF_Au_: area fraction of gold, R_sh_: nanomesh sheet resistance, J_SC_: short circuit current density, V_OC_: open circuit voltage, FF: fill factor and PCE: power conversion efficiency of P3HT:PC_61_BM devices.

**Table 2:** Optimum and average of five (in parenthesis) PTB7:PC_71_BM device parameters.

*P* [nm]	*d* [nm]	AF_Au_ [%]	R_sh_ [Ω/ □]	J_SC_ [mA/cm^2^]	V_OC_ [V]	FF [%]	PCE [%]
ITO reference	—	—	17.4	12.9 (12.8)	0.71 (0.69)	65 (66)	6.0 (5.9)
202	155	46	13.4	7.9 (7.7)	0.69 (0.68)	66 (67)	3.6 (3.4)
375	293	46	8.8	8.7 (8.6)	0.68 (0.68)	66 (67)	3.9 (3.9)
570	462	43	8.5	9.2 (8.8)	0.70 (0.70)	65 (65)	4.2 (4.1)
1040	853	37	13.9	10.2 (9.6)	0.70 (0.69)	65 (65)	4.6 (4.3)
2560	2077	37	11.7	9.3 (9.3)	0.68 (0.68)	67 (63)	4.2 (4.0)

*P*: nanomesh periodicity, *d*: hole diameter, AF_Au_: area fraction of gold, R_sh_: nanomesh sheet resistance, J_SC_: short circuit current density, V_OC_: open circuit voltage, FF: fill factor and PCE: power conversion efficiency of P3HT:PC_71_BM devices.
