# Supplementary material for: Plasmonic nanomeshes: their ambivalent role as transparent electrodes in organic solar cells
Source: Sci Rep. 2017 Feb 15;7:42530. doi: 10.1038/srep42530 (PMC5309773; doi:10.1038/srep42530)
Supplement: Supplementary Information [file srep42530-s1.pdf]

Supplementary Information

Plasmonic nanomeshes: their ambivalent role as transparent electrodes in organic solar cells

Christian Stelling[1], Chetan R. Singh[2], Matthias Karg[3], Tobias König[4]*, Mukundan Thelakkat[2]*, Markus Retsch[1]*

[1]Physical Chemistry – Polymer Systems, University of Bayreuth, Universitätsstr. 30, 95447 Bayreuth, Germany
E-mail: Markus.Retsch@uni-bayreuth.de
[2]Applied Functional Polymers, Macromolecular Chemistry I, University of Bayreuth, Universitätsstr. 30, 95447 Bayreuth, Germany
E-mail: Mukundan.Thelakkat@uni-bayreuth.de
[3]Physical Chemistry I, Heinrich-Heine-Universität, 40204 Düsseldorf, Germany
[4]Institute of Physical Chemistry and Polymer Physics, Leibniz-Institut für Polymerforschung Dresden e. V., Hohe Straße 6, 01069 Dresden, Germany and Cluster of Excellence Centre for Advancing Electronics Dresden (CFAED), Technische Universität Dresden, 01062 Dresden, Germany
E-mail: Koenig@ipfdd.de

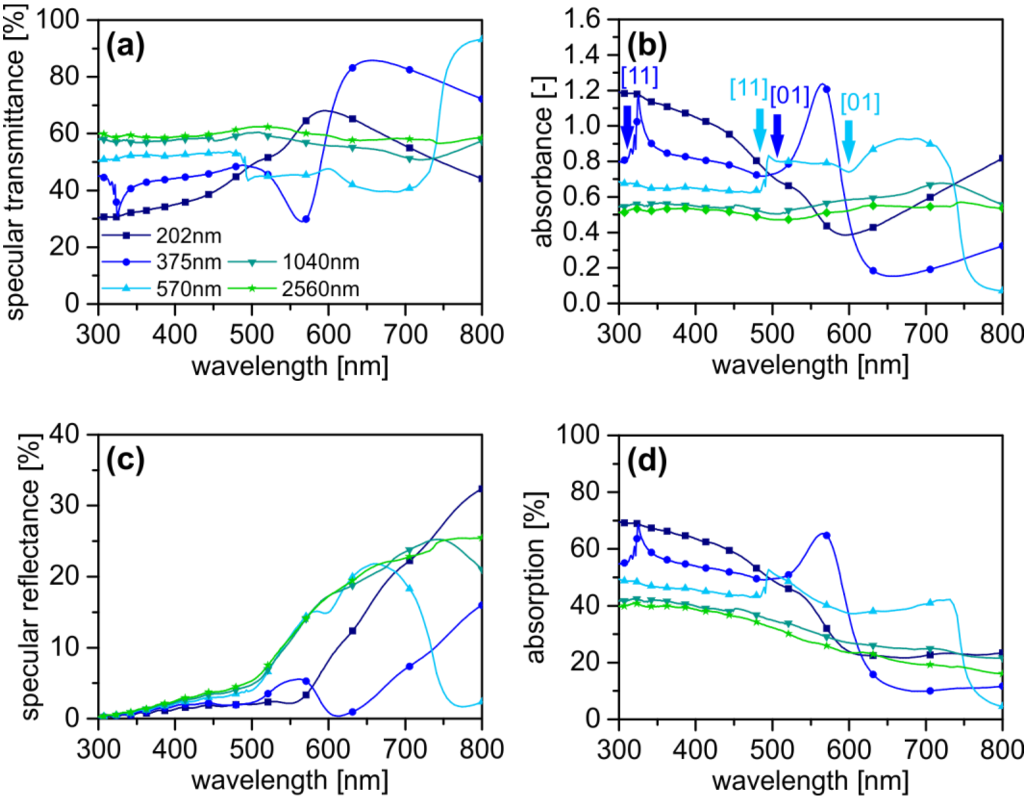

Supplementary Figure S1. FDTD simulation of the pure nanomeshes on glass normalized to the glass substrate with variable periodicity *P* and constant gold area fraction of 40 %. Normal incident specular transmittance (a), absorbance (b), specular reflectance (c) and absorption (d) spectra calculated with unpolarized light. The arrows in (b) indicate the Bragg diffraction modes for P = 375 nm and P = 570 nm.

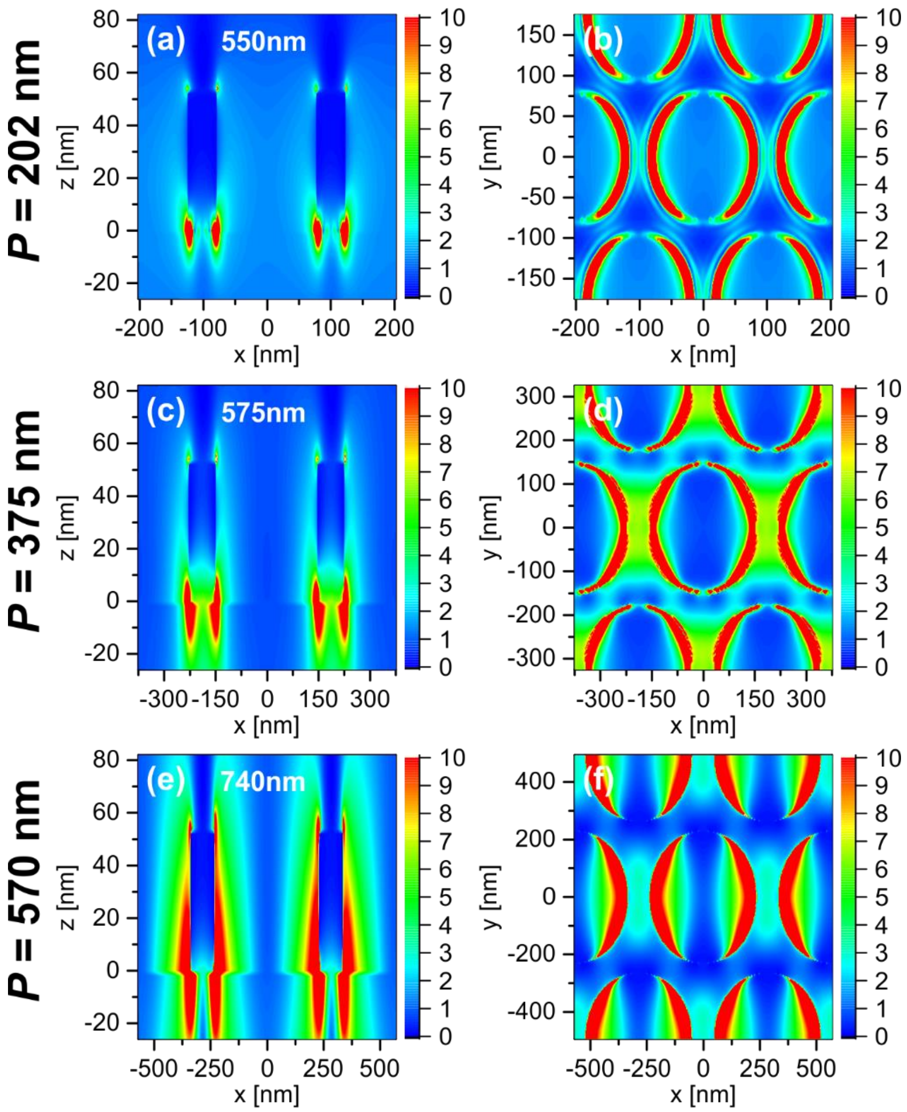

Supplementary Figure S2. Simulated electric field distributions $|E|^2/|E_0|^2$ for the pure nanomeshes with variable periodicity on glass. Cross-section electric field profile (a) and top view electric field profile (b) for *P* = 202 nm and a wavelength of 550 nm. Cross-section electric field profile (c) and top view electric field profile (d) for *P* = 375nm and a wavelength of 575 nm. Cross-section electric field profile (e) and top view electric field profile (f) for *P* = 570 nm and a wavelength of 740 nm.

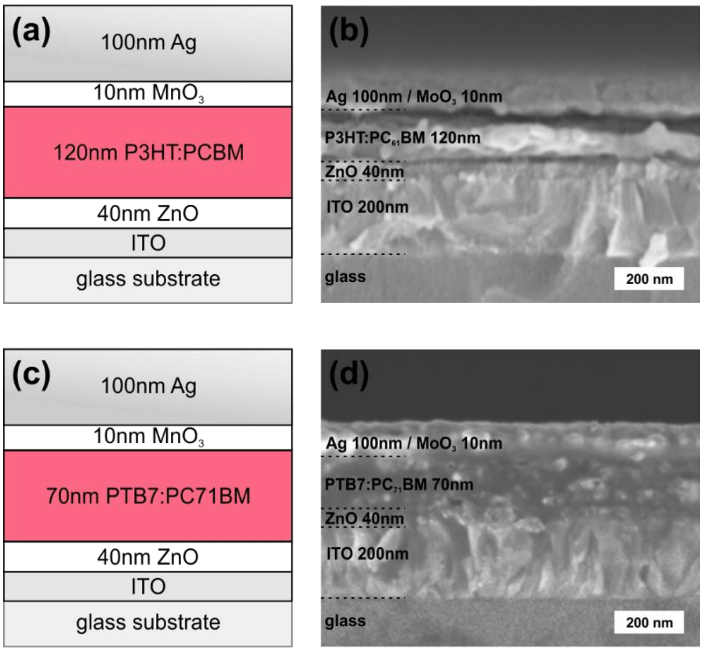

Supplementary Figure S3. ITO reference devices. Schematic illustration of the ITO reference device structure with (a) P3HT:PC$_{61}$BM and (c) PTB7:PC$_{71}$BM active layer. SEM cross-section of the ITO reference device with (c) P3HT:PC$_{61}$BM and (d) PTB7:PC$_{71}$BM active layer.

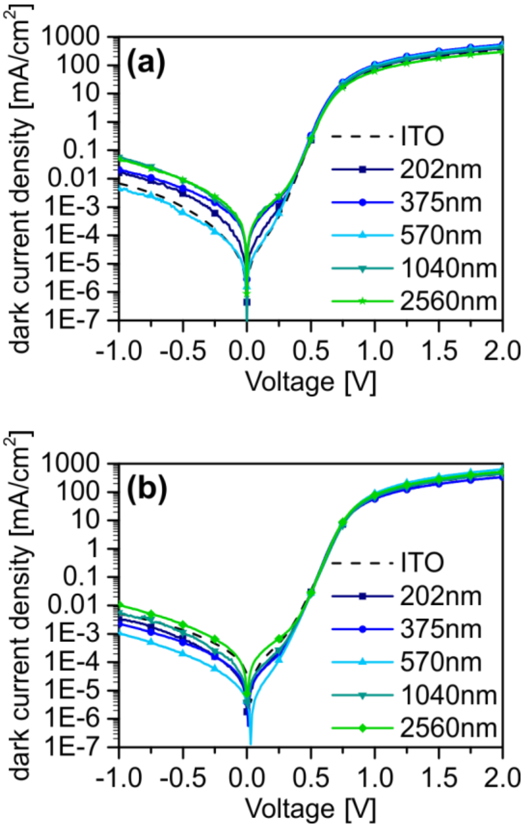

Supplementary Figure S4. Dark current-density characteristics. Dark current-density - voltage curves of (a) P3HT:PC$_{61}$BM and (b) PTB7:PC$_{71}$BM solar cells for different hole-to-hole distances on nanomesh electrode.

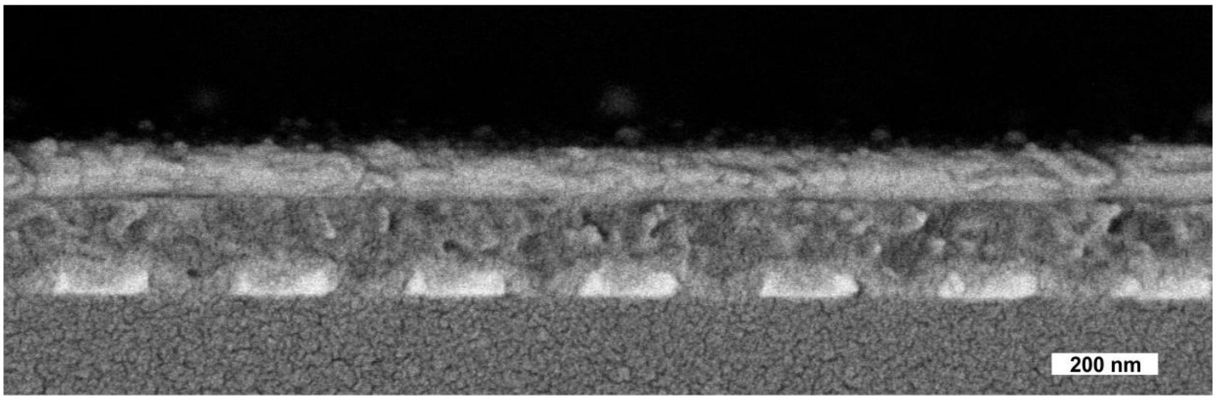

Supplementary Figure S5. BSE image of nanomesh solar cell. SEM cross-section of the nanomesh device with P3HT:$PC_{61}BM$ active layer and $P = 202$ nm measured with the backscattered electron (BSE) detector.

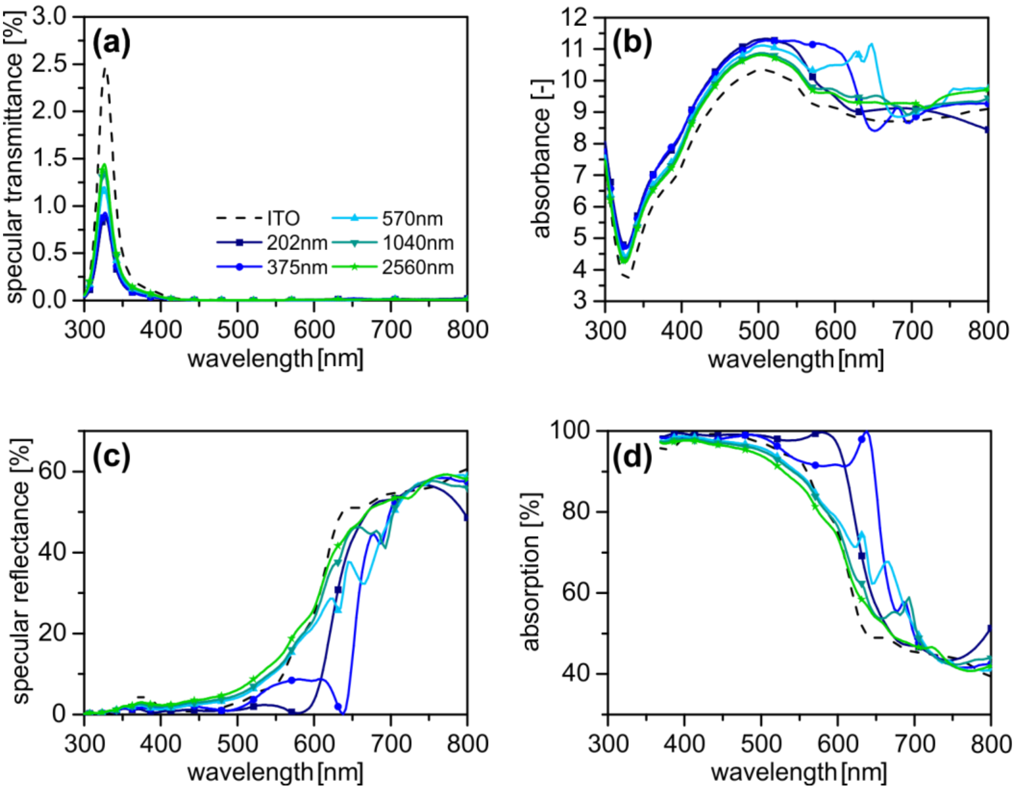

Supplementary Figure S6. FDTD simulation of the P3HT:$PC_{61}BM$ solar cell devices with gold nanohole electrodes and different periodicities compared to ITO reference devices. Normal incident specular transmittance (a), absorbance (b), specular reflectance (c) and absorption (d) spectra.

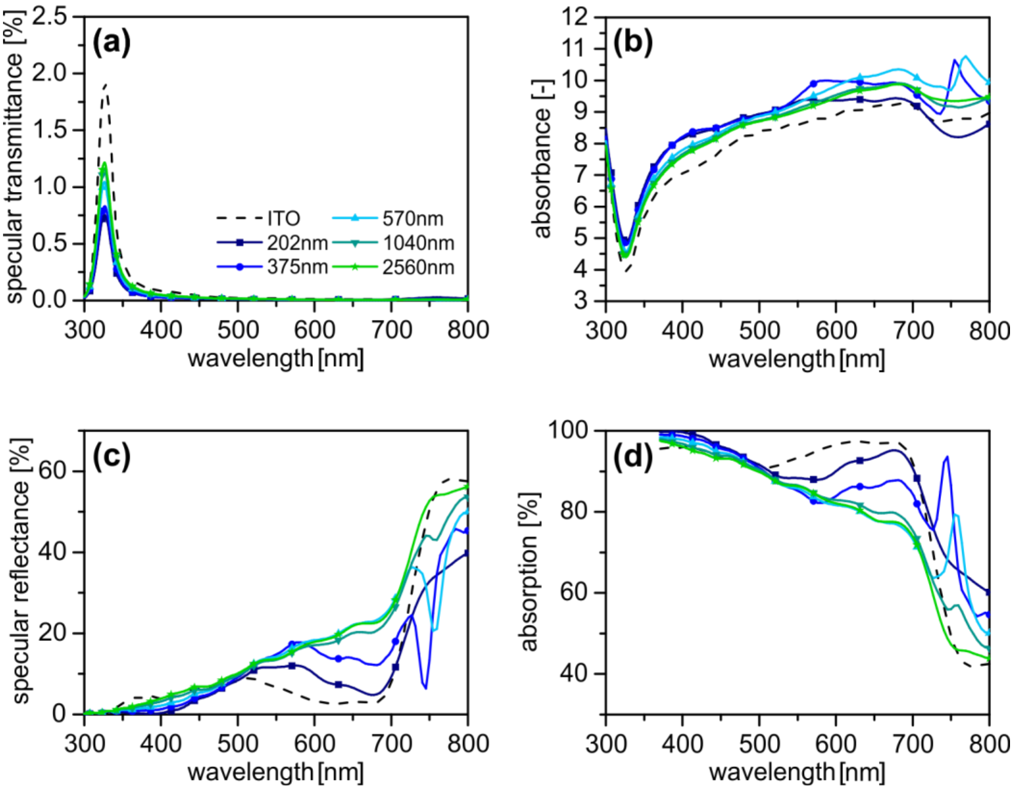

Supplementary Figure S7. FDTD simulation of the PTB7:$PC_{71}BM$ solar cell devices with gold nanohole electrodes and different periodicities compared to ITO reference devices. Normal incident specular transmittance (a), absorbance (b), specular reflectance (c) and absorption (d) spectra.

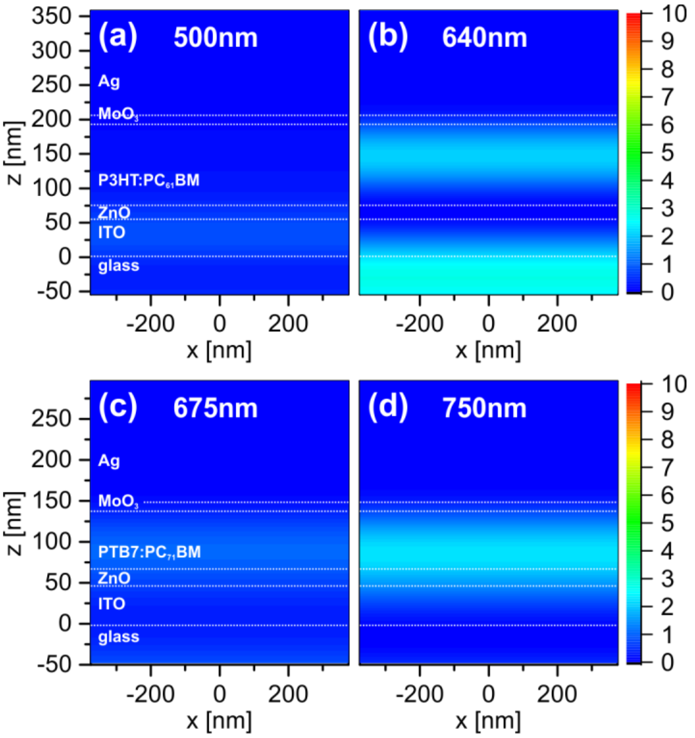

Supplementary Figure S8. Electric field distributions of ITO reference devices. Cross-section electric field distributions $|E|^2/|E_0|^2$ of the ITO reference devices for P3HT:$PC_{61}BM$ at 500 nm (a) and 640 nm (b) and for PTB7:$PC_{71}BM$ at 675 nm (c) and 750 nm (d).

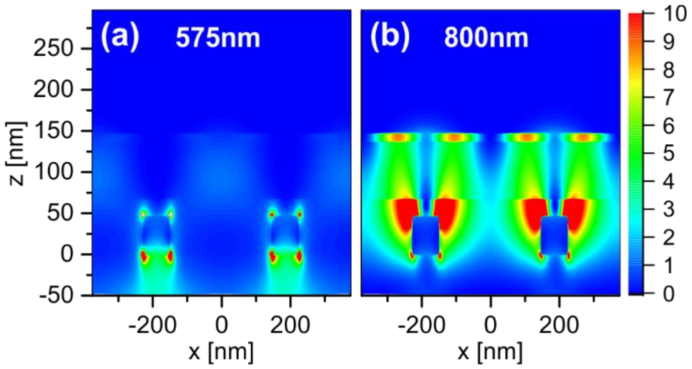

Supplementary Figure S9. FDTD simulation of the PTB7:$PC_{71}BM$ solar cell device with a gold nanomesh electrode and *P* = 375 nm. Cross-section electric field distributions $|E|^2/|E_0|^2$ at 575 nm (a) and 800 nm (b).

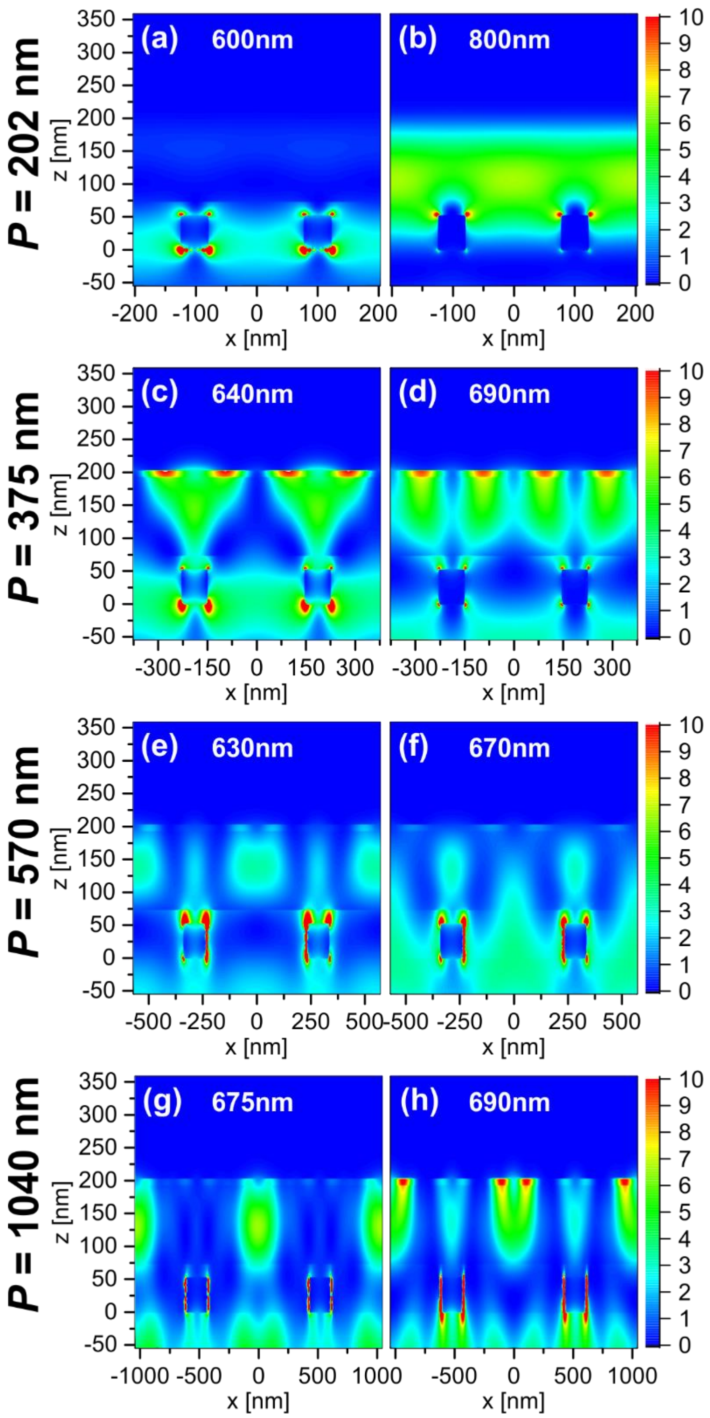

Supplementary Figure S10. FDTD simulation of the P3HT:PC_{61}BM solar cell devices with gold nanomesh electrodes and variable nanomesh periodicities. Cross-section electric field distributions $|E|^2/|E_0|^2$ at 600 nm (a) and 800 nm (b) for P = 202 nm, at 640 nm (c) and 690 nm (d) for P = 375 nm, at 630 nm (e) and 670 nm (f) for P = 570 nm, at 675 nm (g) and 690 nm (h) for P = 1040 nm.

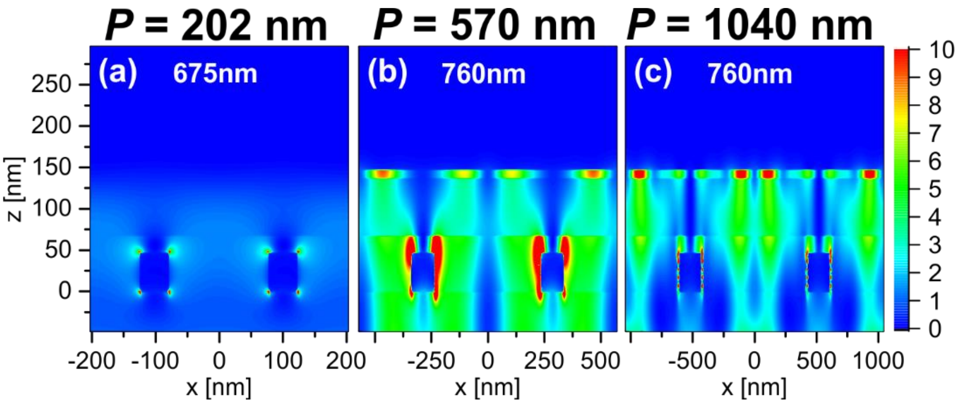

Supplementary Figure S11. FDTD simulation of the PTB7:PC_{71}BM solar cell devices with gold nanomehs electrodes and variable nanomesh periodicities. Cross-section electric field distributions $|E|^2/|E_0|^2$ at 675 nm for P = 202 nm (a), at 760 nm for P = 570 nm (b), at 760 nm for P = 1040 nm (c).

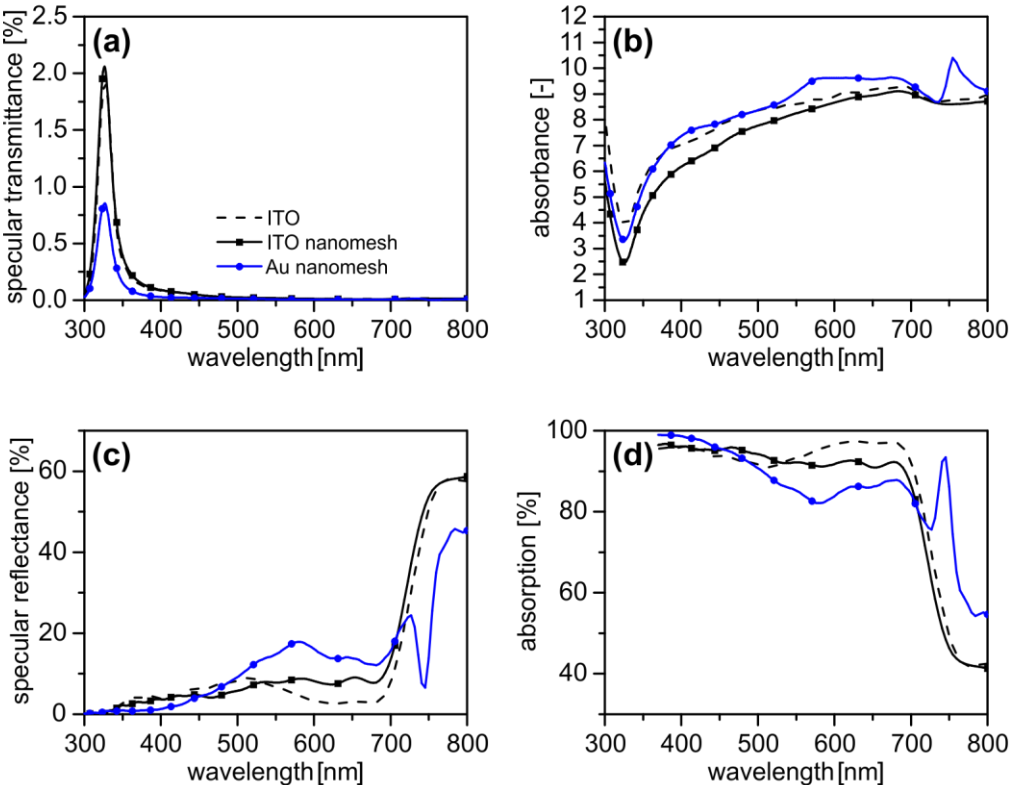

Supplementary Figure S12. FDTD simulation of PTB7:PC_{71}BM solar cell devices with gold nanomesh electrodes and ITO nanomehs electrodes with *P* = 375 nm compared to the planar ITO reference device. Normal incident specular transmittance (a), absorbance (b), specular reflectance (c) and absorption (d) spectra.

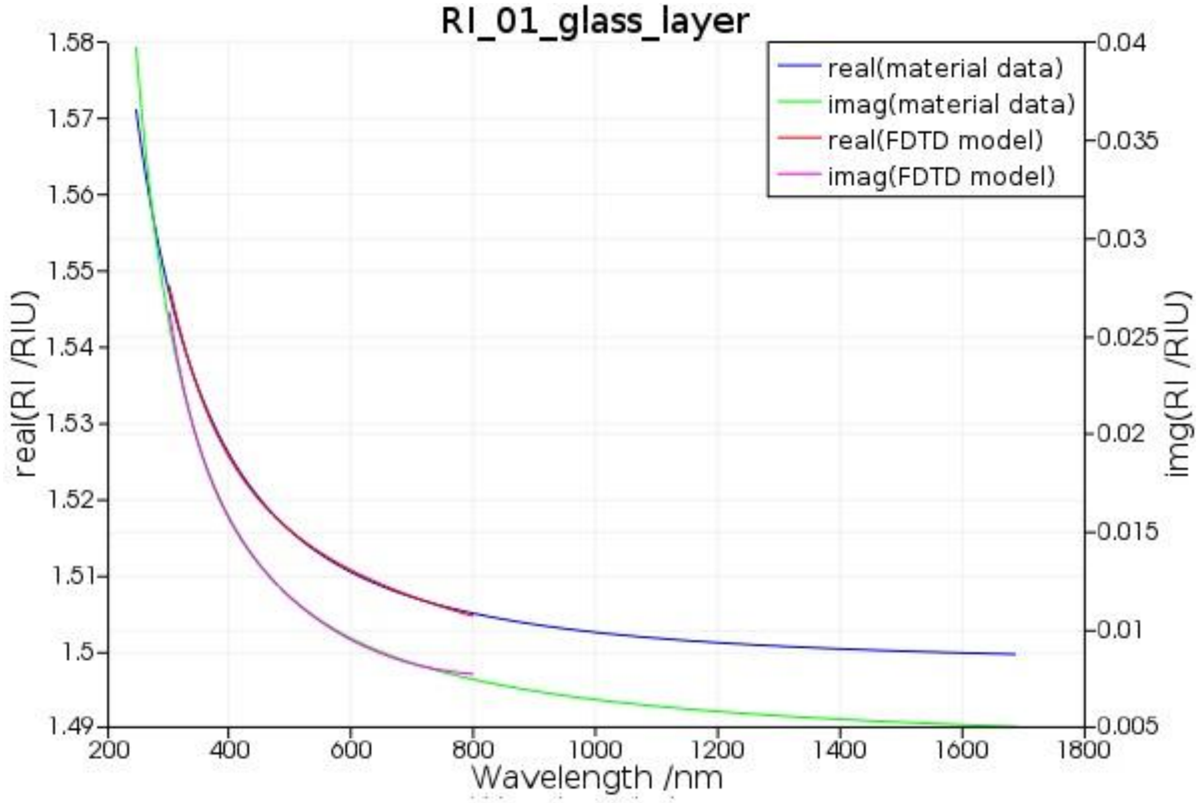

Supplementary Figure S13: Refractive index of glass. Complex refractive index (RI) of the glass layer (standard microscopy slides, *Menzel*, Braunschweig, Germany) determined with spectral ellipsometry (material data) and FDTD approximation with a polynomial function (FDTD model). For further usage the raw data (material data) will be available at http://refractiveindex.info/.

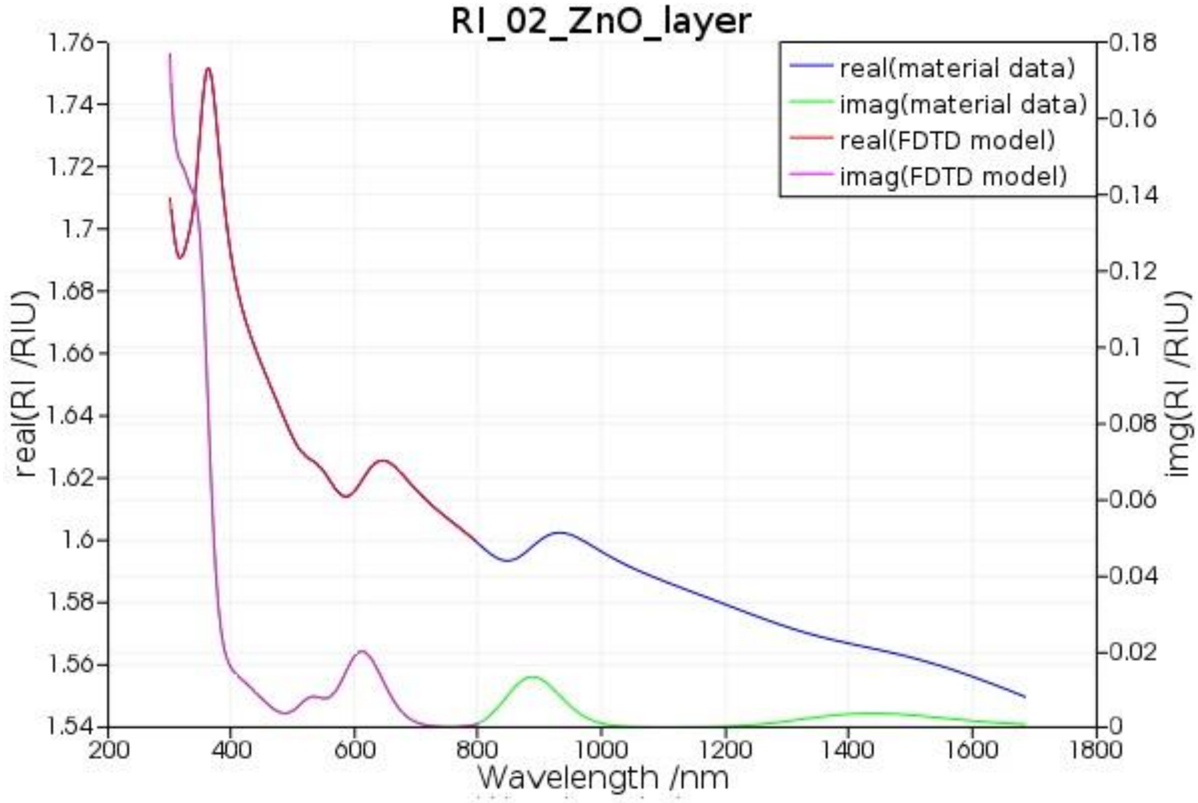

Supplementary Figure S14: Refractive index of ZnO. Complex refractive index (RI) of the ZnO layer determined with spectral ellipsometry (material data) and FDTD approximation with a polynomial function (FDTD model).

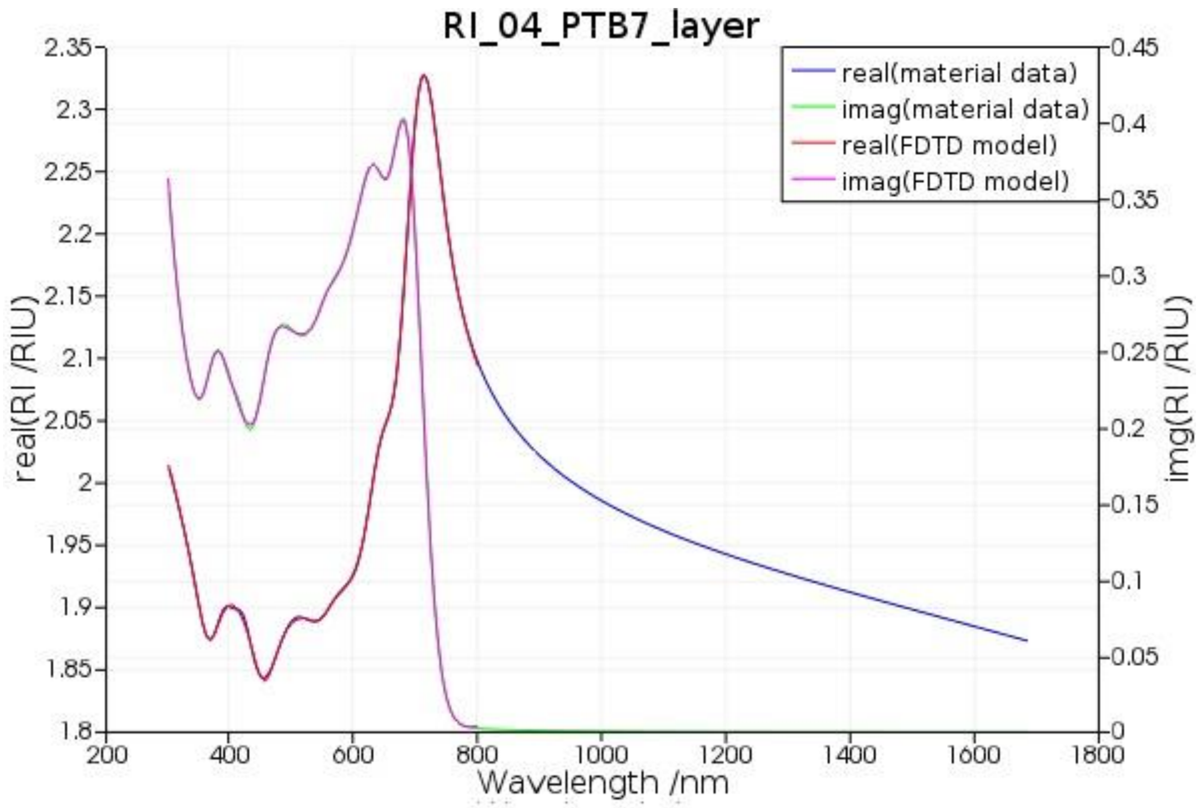

Supplementary Figure S15: Refractive index of PTB7:$PC_{71}BM$. Complex refractive index (RI) of the PTB7:$PC_{71}BM$ layer determined with spectral ellipsometry (material data) and FDTD approximation with a polynomial function (FDTD model).

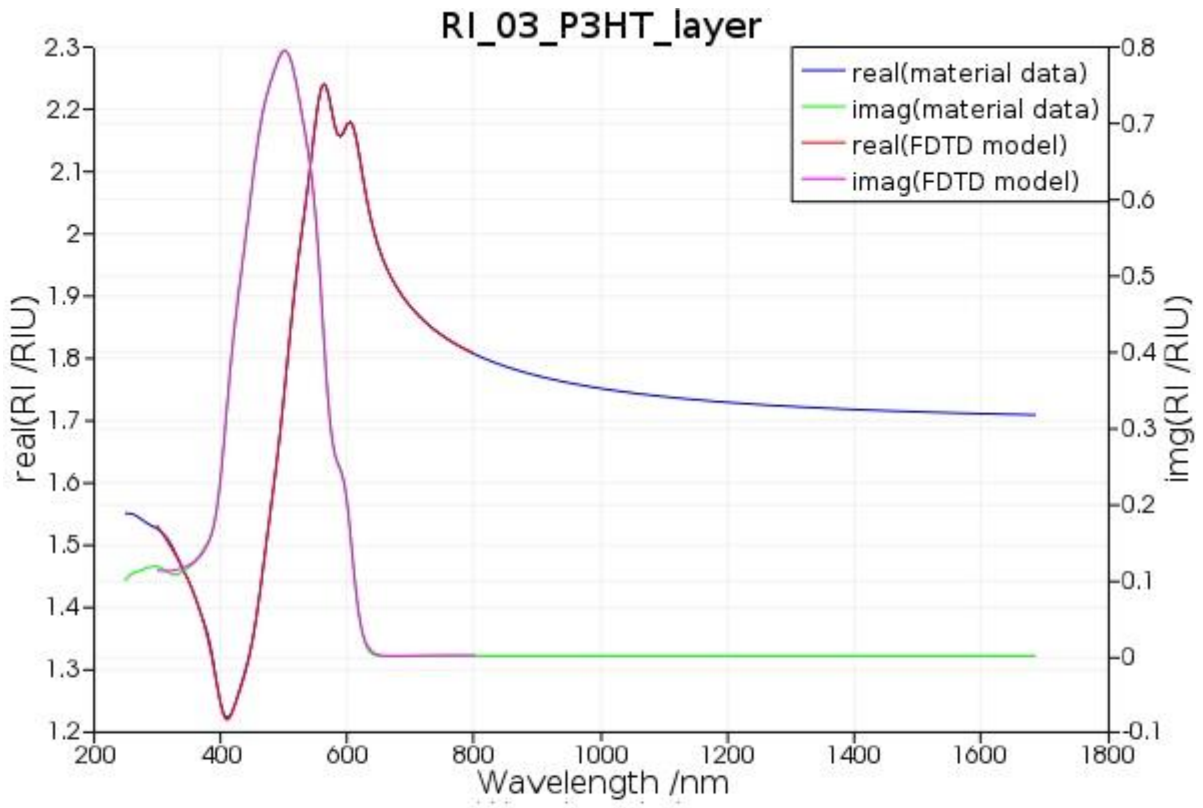

Supplementary Figure S16: Refractive index of P3HT:PC$_{61}$BM. Complex refractive index (RI) of the P3HT:PC$_{61}$BM layer determined with spectral ellipsometry (material data) and FDTD approximation with a polynomial function (FDTD model).

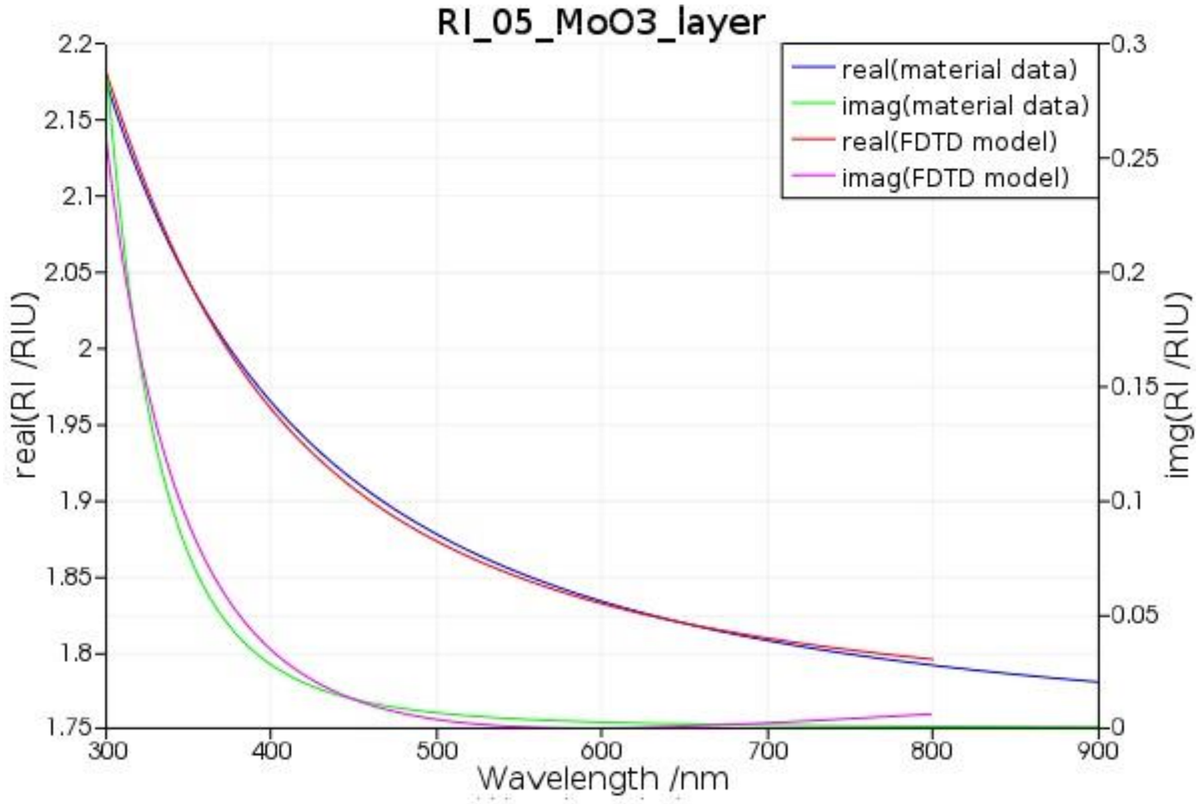

Supplementary Figure S17: Refractive index of MoO_3. Complex refractive index (RI) of the MoO_3 layer determined with spectral ellipsometry (material data) and FDTD approximation with a polynomial function (FDTD model).

Gold was taken from Johnson and Christy (JC) [Johnson, P. B.; Christy, R. W. Optical Constants of the Noble Metals. Phys. Rev. B 1972, 6, 4370-4379.]

Ag was taken from Hagemann et al. (CRC) [Hagemann, H. J.; Gudat, W.; Kunz, C. Optical Constants from the Far Infrared to the X-Ray Region: Mg, Al, Cu, Ag, Au, Bi, C, and A12O3. J. Opt. Soc. Am. A 1975, 65, 742-744.]

ITO from was taken from the CompleteEASE (Version 5.07) refractive index database.
